# Comparative Functional and Phylogenomic Analyses of Host Association in the Remoras (Echeneidae), a Family of Hitchhiking Fishes

**DOI:** 10.1093/iob/obz007

**Published:** 2019-05-10

**Authors:** C P Kenaley, A Stote, W B Ludt, P Chakrabarty

**Affiliations:** 1 Department of Biology, Boston College, Chestnut Hill, MA 02467, USA; 2 Department of Organismic and Evolutionary Biology, Harvard University, Cambridge, MA 02138, USA; 3 School of Marine and Environmental Affairs, University of Washington, Seattle, WA 98105, USA; 4 Smithsonian National Museum of Natural History, Washington, DC 20560, USA; 5 Museum of Natural Science, Ichthyology Section, Department of Biological Sciences, Louisiana State University, Baton Rouge, LA 70803, USA

## Abstract

The family Echeneidae consists of eight species of marine fishes that hitchhike by adhering to a wide variety of vertebrate hosts via a sucking disc. While several studies have focused on the interrelationships of the echeneids and the adhesion performance of a single species, no clear phylogenetic hypothesis has emerged and the morphological basis of adhesion remains largely unknown. We first set out to resolve the interrelationships of the Echeneidae by taking a phylogenomic approach using ultraconserved elements. Then, within this framework, we characterized disc morphology through *µ*-CT analysis, evaluated host specificity through an analysis of host phylogenetic distance, and determined which axes of disc morphological variation are associated with host diversity, skin surface properties, mean pairwise phylogenetic distance (MPD obs.), and swimming regime. We recovered an extremely well-supported topology, found that the specificity of host choice is more variable in a pelagic group and less variable in a reef-generalist group than previously proposed, and that axes of disc morphospace are best explained by models that include host skin surface roughness, host MPD obs., and maximum host Reynolds number. This suggests that ecomorphological diversification was driven by the selection pressures of host skin surface roughness, host specialization, and hydrodynamic regime.

## Synopsis

Análisis Comparativos Funcionales y Filogenómicos de la Asociación de Hospedadores en las Remoras (Echeneidae), una Familia de Peces que Hacen Autostop La familia Echeneidae se compone de ocho especies de peces marinos que hacen autostop al adherirse a una amplia variedad de hospedadores vertebrados a través de un disco de succión. Si bien varios estudios se han centrado en las interrelaciones de los ecenoides y el rendimiento de la adhesión de una sola especie, no ha surgido una hipótesis filogenética clara, mientras que la base morfológica de la adhesión sigue siendo en gran parte desconocida. Primero nos propusimos resolver las interrelaciones de los Echeneidae mediante un enfoque filogenómico utilizando elementos ultraconservados. Luego, dentro de este marco, caracterizamos la morfología del disco mediante análisis de micro-TC, evaluamos la especificidad del huésped mediante un análisis de la distancia filogenética del huésped y determinamos qué ejes de variación morfológica del disco están asociados con la diversidad del huésped, las propiedades de la superficie de la piel, la distancia filogenética media por pares, y régimen de natación. Recuperamos una topología extremadamente bien soportada, encontramos que la especificidad de la elección del huésped es más variable en un grupo pelágico y menos variable en un grupo generalista de arrecifes que lo propuesto anteriormente, y que los ejes del morfoespacio de disco se explican mejor mediante modelos que incluyen rugosidad de la superficie de la piel del huesped, distancia filogenética pareada media del huésped y número de Reynold máximo del huésped. Esto sugiere que la diversificación ecomorfológica fue impulsada por las presiones de selección de la rugosidad de la superficie de la piel del huésped, la especialización del huésped y el régimen hidrodinámico. Translated to Spanish by J. Heras (herasj01@gmail.com)

Análises Funcional Comparada e Filogenômica de Associação de Hospedeiros em Remoras (Echeneidae), uma Família de Peixes Caroneiros A família Echeneidae é composta por oito espécies de peixes marinhos que pegam carona se aderindo a uma grande variedade de hospedeiros vertebrados através de um disco de sucção. Embora vários estudos tenham focado as inter-relações dos echineídeos e o desempenho de adesão a uma única espécie, não há nenhuma hipótese filogenética clara desenvolvida, e a base morfológica da adesão ainda é em grande parte desconhecida. Primeiro, decidimos resolver as inter-relações em Echeneidae adotando uma abordagem filogenômica usando elementos ultraconservados. Em seguida, ainda neste contexto, caracterizamos a morfologia do disco através da análise “μ-CT,” avaliamos a especificidade do hospedeiro através de análise da distância filogenética em relação ao hospedeiro, e determinamos quais eixos de variação morfológica do disco estão associados à diversidade de hispedeiros, propriedades da superfície da pele, média de distâncias filogenéticas pareadas e regime de natação. Nós recuperamos uma topologia com suporte extremamente alto, descobrimos que a especificidade da escolha do hospedeiro é mais variável em grupos pelágicos e menos variável em grupos generalistas recifais do que anteriormente proposto, e que os eixos morfoespaciais do disco são melhor explicados por modelos que incluem a rugosidade da superfície da pele do hospedeiro, a distância filogenética média do hospedeiro e o máximo valor de Reynold também do hospedeiro. Isto sugere que a diversificação ecomorfológica foi impulsionada pelas pressões seletivas sobre a rugosidade da superfície da pele do hospedeiro, especialização do hospedeiro e pelo regime hidrodinâmico. Translated to Portuguese by J.P. Fontenelle (jp.fontenelle@mail.utoronto.ca)

## Introduction

**Fig. 1:**
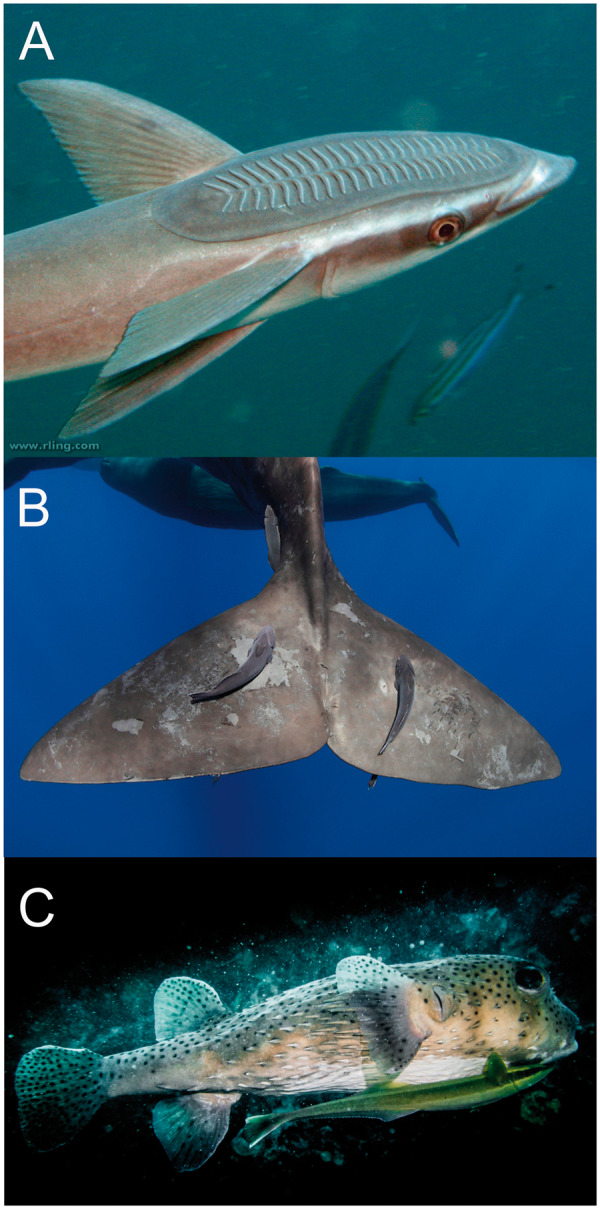
The remora disc system and examples of extreme host choice. The sucking disc of *Echeneis naucrates* in panel (**A**). *Remora australis* on the fluke of a sperm whale (*Physeter macrocephalus*) in panel (**B**) and *E. naucrates* on a porcupine fish (*Diodon* sp.) panel (**C**).

The teleost family Echeneidae, or the remoras, is a group of marine fishes that is most well known for its hitchhiking behavior in which species adhere to a variety of hosts via a remarkably modified dorsal fin (Fig. 1A;Cressey and Lachner 1970; O’Toole 2002; Britz and Johnson 2012). These fishes adhere to a striking variety of vertebrate hosts with diverse behaviors and morphologies, ranging from the body of small reef fishes to the flukes of enormous cetaceans (Fig. 1B,C). It has been proposed that hitchhiking evolved as a means to increase access to food resources, reduce the cost of transport, and provide protection from predators (Muir and Buckley 1967; O’Toole 2002; Beckert et al. 2016). The family comprises three genera and eight species: *Echeneis naucrates*, *E. neucratoides*, *Phtheirichthys lineatus*, *Remora remora*, *R. albescens*, *Remora australis*, *R. brachyptera*, and *R. osteochir*. The Echeneidae has received a considerable amount of attention, including studies of their hydrodynamic load on their host (Beckert et al. 2016), as the basis for bioinspired platforms (Beckert 2016; Wang et al. 2017), the function of their unique cranial vasculature (Flammang and Kenaley 2017), and especially their phylogenetic interrelationships (O’Toole 2002; Gray et al. 2009; Friedman et al. 2013). Despite this, the phylogeny of remoras remains unresolved and the morphological basis of disc adhesion performance has not yet been addressed within a comparative context.

O’Toole (2002) proposed the first phylogenetic hypothesis for the group and concluded that it encompassed two ecologically distinct clades: a reef-generalist group comprised of (*Echeneis naucrates*, *E. neucratoides*, and *P. lineatus*) that have no clear host preference and the pelagic specialists of the genus *Remora* that have strong host preferences. *Remora brachyptera*, *R. osteochir*, and *R. remora* attach primarily to pelagic sharks, billfishes (family Istiophoridae), and swordfish (*Xiphias gladius*). *Remoraalbescens* appears to have an obligate relationship with mantas (genus *Manta*) while *R. australis* attaches exclusively to cetaceans (Cressey and Lachner 1970; O’Toole 2002).

**Fig. 2:**
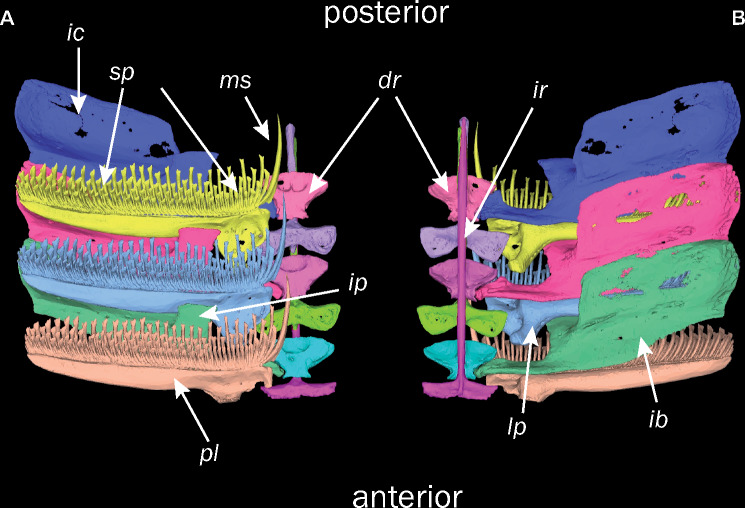
Osteological components of the remora sucking disc as represented by *Remora osteochir*. Dorsal view in panel (**A**) and ventral view in panel (**B**) of lamellae 13–15 from the right side of the disc (MCZ10128). dr., distal radial; ic, intercalary bone; ip, intercalary bone process; ir, interneural ray; lp, lamella process; ms, medial spine; pl, pectinated lamellae; sp, spinules.

The osteology of the remora disc structure is astonishingly complex (Fig. 2 and Supplementary Fig. S1) (Fulcher and Motta 2006; Britz and Johnson 2012). Recently, Britz and Johnson (2012) confirmed earlier hypotheses (de Blainville 1822; Voigt 1823) that the disc develops as rearrangements of the dorsal-fin spines and radials. These elements form as many as 26 bilaterally arranged pectinated lamellae (Fig. 1A and Supplementary Fig. S1). The dorsal surface of the lamellae supports spinules, rows of thin, bony elements that serve as the point of contact with the host surface (Fig. 2; Fulcher and Motta 2006). At its medial margin, each lamella bears an elongated medial spinule (Fig. 2). Near the proximal ventral aspect of each lamella sits a long process that projects anteriorly (Fig. 2). Ventral to the pectinated lamellae, large intercalary bones, homologs of the distal radials in other teleosts (Britz and Johnson 2012), interlock with one another and with the lamellae (Fig. 2). A posterior process on the intercalary bone projects dorsally (Fig. 2) and, with the lamella process, form an interlocking disc structure and linkage system. Located medial to the lamellae is the interneural ray, a homolog of the proximal-middle radial (Britz and Johnson 2012). This structure is characterized by a wide dorsal head and an elongated spine extending ventrocaudally (Fig. 2).

The contribution of any aspects of this system to adhesion performance has received very little attention. Fulcher and Motta (2006) found that dead specimens of *E. naucrates* produced greater suction pressure on acrylic glass than on shark skin; however, considerably more posteriorly directed force was required to dislodge specimens from shark skin. Later, Beckert et al. (2015) confirmed through computation methods the importance of spinules in generating friction and shear resistance. These studies have focused on a single species and have not addressed variation in host morphology or behavior that may require complementary variation in disc anatomy for effective adhesion. Given the considerable interspecific variation in disc morphology of remoras (Stote and Kenaley 2014) and broad range of host choice, many other aspects of disc osteology may also contribute substantially to adhesion performance over a wide spectrum of host body surfaces. A comparative approach that considers the major axes of disc variation and important host properties may elucidate which components of disc morphology contribute to high-performance adhesion.

To this end, we first set out to resolve the interrelationships of the Echeneidae by taking a phylogenomic approach using ultraconserved elements (UCEs) in the hopes that hundreds of loci from across genomes would provide a stronger phylogenetic signal than the few loci used in previous analyses. UCEs have proven valuable in generating robust phylogenetic hypotheses in a variety of vertebrate groups at different taxonomic levels (McCormack et al. 2012; Harrington et al. 2016). We then sought to reassess host specificity in all remora species through a characterization of host phylogenetic breadth, establish major axes of remora disc variation, and assess in a comparative framework which components of the disc are associated with host phylogenetic diversity, skin morphology, and host swimming regime.

## Materials and methods

### Host diversity

We compiled host records from O’Toole (2002) and by querying museum records through the Global Biodiversity Information Facility (GBIF) with the “rgbif” package (Chamberlain et al. 2018) written for R (R Core Team 2017) (Supplementary Table S1). From these data, we characterized host diversity based on mean pairwise phylogenetic distance (MPD obs.) and MPD with standardized effect size (MPD obs. *z*) (Webb et al. 2002) among all hosts for each remora species using the R package “picante” (Kembel et al. 2010). MPD obs. *z* values higher than zero indicate phylogenetic evenness (species more distantly related than expected) and host-choice generalism, whereas MPD SES values lower than zero indicate phylogenetic clustering (species more closely related than expected) and high host specificity. We obtained two-tailed *P* values by comparing the observed MPD values (MPD obs.) with those from the 1000 randomized hosts distributions. For MPD analysis, we compiled a concatenated matrix of five genomic loci from GenBank, including *COI*, *IRBP*, *ENC1*, *RAG1*, and *rhodopsin* (Supplementary Table S2). For any loci not available for a given host in GenBank, we downloaded a sequence from that species’ congener or, if unavailable, a confamilial species. The final host matrix was 4271 bp. We used RAxML (Stamatakis 2014) analysis with 100 rapid bootstraps using the GTRGAMMA model of rate heterogeneity with *Petromyzonmarinus* as the outgroup to produce the consensus tree in Fig. 4. This tree was largely congruent with current hypotheses of vertebrate interrelationships at the ordinal level (Alfaro et al. 2009; Steeman et al. 2009; Naylor et al. 2012; Near et al. 2012; Betancur-R et al. 2013) with the exception of the Percomorpha in that our representatives of the Carangimorphariae (jacks, billfishes, and barracuda) were recovered as sister to the Lutjanidae. Following Betancur-R et al. (2013) and Near et al. (2012), we manually placed the Carangimorphariae as sister to all the percomorphs minus the scombroids. To visualize the phylogenetic distribution of host choice, we integrated our host association dataset, the host tree, and the phylogenetic hypothesis for the echeneids to produce a tanglegram of remora host choice.

### Remora phylogeny

To confidently determine the remora interrelationships, we used a targeted capture approach to recover UCEs throughout the genome (Faircloth et al. 2012). This approach recovers conserved regions shared across certain taxa and uses variable flanking regions for phylogenetic analyses. UCEs have been highly informative for a variety of actinopterygian groups (Faircloth et al. 2013; Alfaro et al. 2018; Burress et al. 2018), including carangimorphs, a higher-level taxon that includes the Echeneidae (Harrington et al. 2016). High-quality genomic DNA was extracted from tissue samples of all extant remora species, as well as two carangid outgroups (*Carangoides armatus* and *Trachinotus blochii*), using a Qiagen DNeasy blood and tissue extraction kit. Genomic DNA was quantified using a Qubit 2.0 fluorometer with a dsDNA broad-range assay kit and approximately 1 µg of DNA from each sample was then sonicated to reduce the samples to an average of 600 bp.

We then prepared Illumina libraries for each sample using a Kappa Hyper Prep Kit (Kappa Biosystems) with dual-indexing barcodes from Faircloth and Glenn (2012). All reactions followed the manufacturers’ protocols, except that reactions were scaled to half the volume recommended. Libraries were amplified using 10 PCR cycles and quantified and pooled in equimolar ratios. This pool was then enriched for UCE loci using the myBaits UCE 0.5k actinopterygian capture kit (Arbor Biosciences). Enrichments followed manufacturers protocols but with 16–18 PCR cycles. Samples were then sequenced at the University of Oklahoma Medical Research Foundation on a PE150 Illumina HiSeq 2500 sequencer.

De-multiplexed samples were trimmed of barcodes and low-quality base calls using trimmomatic (Bolger et al. 2014), as part of the Illumiprocessor wrapper (Faircloth 2015). We assembled UCE loci *denovo* with the program Trinity (Grabherr et al. 2011) using default settings. After assembly, we also added the putative sister taxa *Coryphaena hippurus* and *Rachycentron canadum* (Johnson 1984; O’Toole 2002; Gray et al. 2009; Friedman et al. 2013) from Alfaro et al. (2018) to our dataset. We then used the PHYLUCE pipeline (Faircloth 2015) to align this dataset with MAFFT (Katoh and Standley 2013), using internal trimming with Gblocks (Castresana 2000; Talavera and Castresana 2007) to construct a 75%-complete data matrix. We then determined an optimal partitioning scheme for this dataset following procedures outlined in Tagliacollo and Lanfear (2018). Briefly, each locus was first partitioned into three sections corresponding to the left and right flanks, and the core using a maximum entropy model. These partitions were then analyzed in PartitionFinder 2.1.1 using the RAxML and “rcluster” options to determine the optimal number of partitions (Lanfear et al. 2014, 2016). Phylogenetic relationships were estimated using RAxML on the CIPRES portal (Miller et al. 2010) using the optimal partitioning scheme and a GTRGAMMA model for all partitions with 1000 rapid bootstrap replicates. An alternative approach using a coalescent quartet-based methods was implemented using SVDquartets (Chifman and Kubatko 2014) in the program PAUP* v4b10 (Swofford 2003). Additionally, we estimated divergence times for the Echeneidae using penalized maximum-likelihood (Sanderson 2002) with the *chronos* function in the R package “ape” (Popescu et al. 2012). We calibrated our phylogeny with two calibration points following Harrington et al. (2016) using a relaxed-clock model that allowed for rate variation and a lambda value of 0. One calibration point constrained the age of Echeneidae with an undetermined fossil species of *Echeneis* (Micklich et al. 2016; see justification for Calibration 6 in Harrington et al. 2016), and the second constrained the root of the phylogeny with the corresponding node and 95% HPD in Harrington et al. (2016).

### Suction disc morphospace

We acquired three preserved individuals of each of the eight remora species for a total sample size of 24 specimens (Supplementary Table S3). We scanned individual heads with a SkyScan1173 high-energy spiral-scan µ-CT unit. Parameter values for amperage, voltage, exposure time, and image rotation varied between 40–136 µA, 51–130 kV, 337–730 ms, and 0.06–0.07°, respectively. This produced voxel sizes ranging from 13.86 to 71.05 µm. We performed slice reconstruction in NRecon (Micro Photonics), and segmentation and volume rendering in Mimics 15.0 (Materialise). An example µ-CT reconstruction is presented in Supplementary Fig. S1.

Morphometric analysis was performed in Mimics with the following protocol. First, the length of the disc was measured from the first to last pectinated lamellae. Disc length was divided into equal fifths to identify five lamellae and associated components for analysis. Based on these five lamellae, we calculated species means of the following morphological measurements from each of the selected areas on the left side of the disc: lamella angle of insertion relative to disc midline, lamella length and width, medial-spine length, interneural-ray length and width, length of lamella process, intercalary-bone length and width, length of the intercalary cup, and medial and lateral-edge lengths of the intercalary bone process (Fig. 2 and Supplementary Fig. S2). We also measured the length of all spinules on lamellae in the first, third, and fifth divisions of the disc. All linear measurements were standardized and size-corrected by dividing these values by specimen standard length. These data were used to define a set of morphometric variables given in Supplementary Table S6 for our estimation of disc morphospace.

We performed phylogenetic principal component analysis (pPCA) (Revell 2009) using a Brownian-motion correlation structure on mean log_10_-transformed disc measurements for each species using the R package “phytools” (Revell 2012). We then constructed a phylomorphospace (Sidlauskas 2008) based on the first, second, and third PCs to visualize the phylogenetic pattern of disc anatomical characters.

### Host skin surface analysis

We acquired one to four samples from 13 common host species listed in Supplementary Table S5 for a total of 27 samples (Supplementary Table S4). Original samples were excised from midlateral portions of the host body and ranged in size from approximately 50 to 150 cm^2^. Samples were sourced from Woods Hole Oceanographic Institute, the Virginia Institute of Marine Sciences (VIMS), the Museum of Comparative Zoology (MCZ), and Boston Sword and Tuna, a local commercial fish wholesaler. The samples acquired from the MCZ and VIMS were excised from preserved specimens, while the remainder of the samples were acquired from recently dead or frozen specimens. During excision, we were careful to remove the skin and some portion of the underlying tissue (either muscle or blubber). This resulted in very little deformation of the skin. We made negative molds of each sample measuring approximately 20 cm^2^ using a synthetic molding material with a maximum resolution of 1 µm (Struer’s RepliSet System). Molds were sputter coated with 10 nm of gold and scanned with a CCI HD optical profilometer using a 20× objective over a scan area of 4 mm× 4 mm. 3D surfaces were reconstructed with TalyMap Platinum 6.2 profiling software. From these profiles, we computed arithmetic mean surface height (*S*_a_ in µm) and kurtosis (*S*_ku_). *S*_a_ is an absolute measure of the height of surface asperities compared with the arithmetical mean height of the surface. *S*_ku_ characterizes the *x*–*y* spread of the height distribution such that a value of 3 indicates a Gaussian 2D profile, while those below 3 and above 3 indicate a rounded and spiked profile, respectively. *S*_a_ and *S*_ku_ values for an additional five hosts were obtained from D. Wainwright (personal communication) using methods described in Wainwright et al. (2017).

### Host swimming regimes

In addition to producing sub-ambient pressure for adhesion to surfaces of variable textures, the echeneid disc system must also resist shear forces due to hydrodynamic drag (Beckert et al. 2016). These forces will vary according to the flow regime surrounding the host. We thus calculated the estimates of each hosts’ maximum Reynolds number (Re), the ratio of inertial to viscous forces as determined by the length of the host and its swimming speed:

(1) Re=LmaxUmaxv,

where *L*_max_ is the maximum host length (in m), *U*_max_ the maximum sustained swimming velocity (in m s^−1^), and *v* the kinematic viscosity of seawater (1.044×10^6^ m^2^ s^−1^). *L*_max_ values were retrieved from FishBase and SeaLifeBase using the “rfishbase” package (Boettiger et al. 2012). We estimated *U*_max_ using Domenici’s (2001) scaling model that was based on data from over 40 aquatic vertebrates, ranging from small teleosts to the largest aquatic vertebrate, the blue whale (*Balaenoptera musculus*):

(2) $$U_{\max}=e^{0.49\;\text{log}\;L+0.60}.$$

Length and speed estimates for each species are presented in Supplementary Table S5.

### Comparative disc–host relationships

To assess which components of disc morphospace are correlated with measures of MPD obs., *S*_a_, *S*_ku_, and maximum Re, we constructed ordinary linear models of all combinations of these variables and each of the first three phylogenetic PCs. Models were compared using corrected Akaike information criterion weights (wAICc). Re values were log_10_ transformed to mitigate the effect of the wide range of values that span several orders of magnitude.

## Results

### Echeneid relationships

Raw reads from the UCE sequencing ranged from 135,536 to 5,045,197 per sample, with an average of 1,482,590. This resulted in an average of 426 UCE loci per species (range of 375–457). Our 75% complete matrix contained 445 loci, totaling 316,162 bp with an average locus length of 703 bp.

**Fig. 3:**
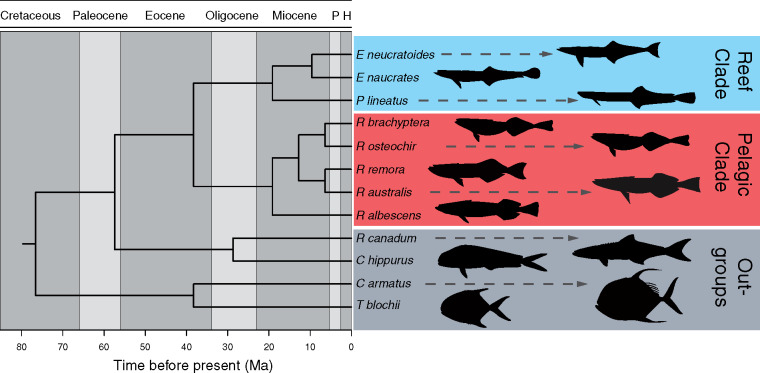
Time-calibrated phylogenetic interrelationships of the Echeneidae based on a topology from an 75%-complete data matrix that included 445 loci. All nodes in the topology had RAxML bootstrap values of 100.

The optimal partition scheme supported 316 partitions. Maximum likelihood and quartet-based analyses resulted in identical relationships and the tree inferred from the 75%-complete dataset shown in Fig. 3 had bootstrap values for all nodes of 100. We recovered a monophyletic Echeneidae comprising two clades: a reef group containing *P. lineatus* sister to *E. naucrates *+ *E. neucratoides* and the other, a pelagic clade, containing all members of the genus *Remora* (Fig. 3). The remora clade consists of *R. albescens* sister to all other species of *Remora* arranged in two sister clades of *R. osteochir *+ *R. brachyptera* and *R. australis *+ *R. remora*. Dating estimates support an origin for the family in the middle to late Eocene (∼38 Ma) and the emergence of the reef and pelagic clades in the early Miocene (∼19 Ma). Both the pelagic and reef clades diversified across the Miocene with all species represented by the end of the Miocene.

### Echeneid host diversity

**Fig. 4:**
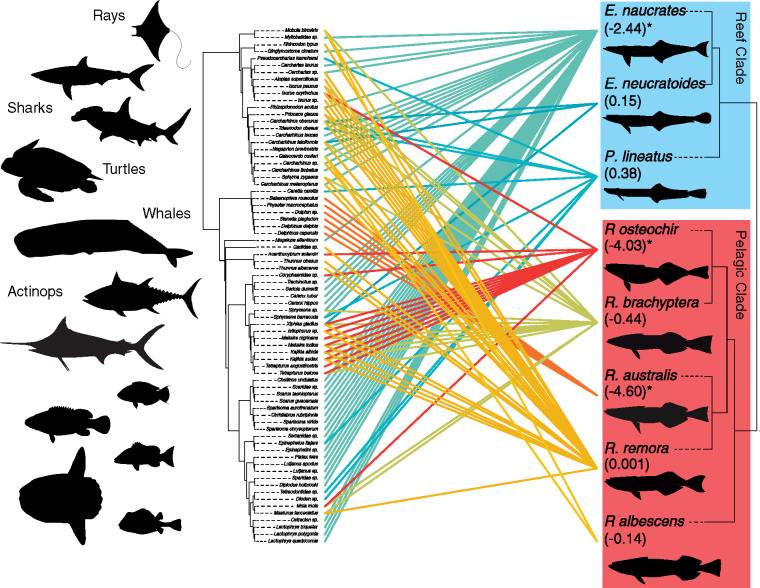
Cophylogenetic tanglegram of echeneid–host associations. Values under echeneid species names represent MPD with standardized effect size (MPD obs. *z*) (Webb et al. 2002). Asterisks indicate significant MPD obs. *z* at the *P* = 0.05 level.

The echeneids adhere to a wide range of marine hosts across the vertebrate tree of life, from sharks to rays, whales, and sea turtles, to a diverse group of actinopterygians (Fig. 4). Two of the three reef species, *E. neucratoides* and *P. lineatus*, had observed MPD obs. *z* values close to 0, indicating that these species demonstrate no phylogenetic clustering in host choice (*P* = 0.38 and 0.55, respectively; Supplementary Table S7 and Fig. 4). Despite associations with several clades of hosts, *E. naucrates* demonstrates significant host clustering with an MPD obs. *z* value of −2.49, the third most extreme value among the eight species (*P* = 0.02; Supplementary Table S7 and Fig. 4). Within the pelagic clade, *R. australis* and *R osteochir* had MPD obs. *z* values well under zero at −4.55 (*P < *0.01) and −4.26 (*P < *0.01), respectively (Supplementary Table S7 and Fig. 4), indicating significant host clustering. However, the remaining three pelagic species had MPD obs. *z* values close to 0 (−0.02 to −0.42, all *P > *0.28; Supplementary Table S7 and Fig. 4), indicating no host specificity.

### Disc morphospace

**Fig. 5:**
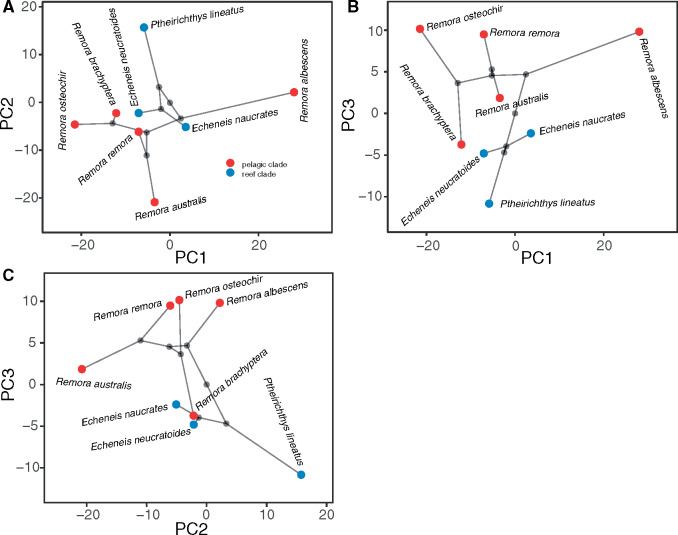
A morphospace of all eight echeneid species that superimposes the branching patterns of the phylogeny (light gray lines) on the plot of the first two (**A**), first and third (**B**), and (**C**) second and third PC axes from the phylogenetic PCA. Principle component 1, PC2, and PC3 account for 45%, 25%, and 16% of disc morphological variance, respectively. PC1 loads heavily for medial-spinule, lamella processes, intercalary cup, intercalary process, interneural ray, and spinule lengths, as well as, width of the head of the interneural ray. Principle component 2 loads heavily for disc length, lamella count, and intercalary bone and lamella aspect ratios. Principle component 3 loads heavily for spinule length range in the lamellae of the mid-section and posterior of the disc.

A summary of results for phylogenetic PCA is presented in Table 1 (comprehensive results in Supplementary Table S6). Principle component 1, the primary axis of morphological variation, explains approximately 45% of the total variance. This represents a synthetic measure of osteological variation in echeneids as medial spinule length, lamella processes length, intercalary cup length, intercalary process length, width of the head of the interneural ray, interneural ray length, and spinule lengths are all highly correlated with PC1. Principle component 2 explains 25% of the disc variation and loads heavily for disc length, lamella count, and intercalary bone and lamella aspect ratios. Principle component 3 accounts for 16% of the variance and loads heavily for spinule length range in the lamellae of the mid-section and posterior of the disc. Together, these three PCs account for a total of 86% of disc morphological variation. A projection of the first three PCs in phylomorphospace (Fig. 5) indicates that the pelagic and reef clades occupy dispersed and overlapping partitions of disc morphospace according to PC1 and PC2 and PC2 and PC3; however, the reef and pelagic clades occupy distinct regions of morphospace defined by PC1 and PC3.

**Table 1:** Summary of phylogenetic PCA analysis of disc morphometric characters with eigenvector coefficients greater than 0.7

	PC1	PC2	PC3
Eigenvalue	10.3	5.7	3.7
Proportion of variance	0.45	0.25	0.16
Cumulative proportion	0.45	0.70	0.86
*Eigenvector coefficients*			
Lam. process length	−0.84	0.13	−0.43
Lam. count	−0.04	−0.97	−0.02
Med. spinule length	−0.96	0.00	−0.03
Disc length	−0.52	−0.78	0.33
Interc. AR	−0.10	0.89	−0.13
Interc. cup width	−0.75	−0.60	0.03
Interc. process length	−0.98	0.16	0.09
Int. ray head width	−0.80	−0.56	−0.07
Int. ray length	−0.96	−0.08	0.20
Lam. AR	−0.20	0.98	−0.02
Ant. spinule length range	−0.15	0.14	0.75
Mid. spinule length range	−0.20	0.04	0.92
Post. spinule length range	−0.27	0.21	0.77
Ant. spinule density	0.90	−0.19	0.39
Mid. spinule density	0.83	−0.24	0.26
Post. spinule density	0.90	−0.26	0.27
Ant. spinule mean length	−0.80	0.45	−0.09
Mid. spinule mean length	−0.93	0.26	0.23
Post. spinule mean length	−0.90	0.19	0.33

See also Supplementary Table S6.

### Comparative disc–host relationships

**Fig. 6:**
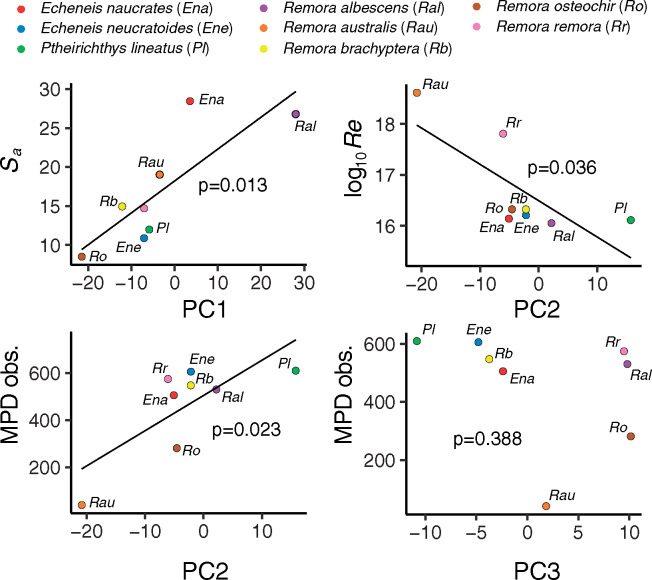
Ordinary linear regressions of phylogenetic PCA eigenvectors (PC1–3) versus important host properties as determined by AICc model fitting. See also Table 2.

Comparison of ordinary linear models of pPCA components revealed that, for PC1, a model that incorporated only *S*_a_ fit best and was significant (wAICc = 0.91, *P* = 0.013; Table 2 and Fig. 6). For PC2, a model that included host MPD obs. fit best and this relationship was significant (wAICc = 0.46, *P* = 0.023); in addition, another model that included only host maximum Re had a similar level of AIC support and was also significant (wAICc = 0.41, *P* = 0.036; Table 2 and Fig. 6). For PC3, a model that included MPD obs. was the best fitting model but this was not significant (wAICc = 0.29, *P* = 0.388; Table 2 and Fig. 6).

**Table 2:** Summary of model fits for linear regressions of pPCA components 1–3 versus host surface roughness (*S*_a_ and *S*_ku_), MPD obs., and estimated maximum Reynolds number (R_e_)

	Model	dAICc	wAICc	*P*-value	Rank
PC1*∼*	*S*_a_	0.00	0.91	0.013	1
	*S*_ku_	7.39	0.02	0.418	2
	MPD obs.	8.27	0.01	0.546	3
PC2*∼*	MPD obs.	0.00	0.46	0.023	1
	Re	0.23	0.41	0.036	2
	*S*_ku_ * *+MPD obs.	4.92	0.04	0.627	3
PC3*∼*	MPD obs.	0.00	0.29	0.388	1
	Re	0.19	0.26	0.504	2
	*S*_ku_	0.50	0.23	0.647	3

Only the top three models according to wAICc are shown.

## Discussion

### Remora phylogenomics

Using a novel UCE dataset that included 445 loci from across the genome, we established a strongly supported phylogenetic hypothesis for the Echeneidae by recovering a monophyletic Echeneidae and monophyletic reef and pelagic groups. We have therefore resolved a long-standing incongruence between the previous hypotheses that were based on morphological data and sequencing of a limited number of loci (O’Toole 2002; Gray et al. 2009; Friedman et al. 2013). The total-evidence (mtDNA + morphology) of Friedman et al. (2013) hypothesized remora interrelationships that are identical to those recovered in our study. This topology differs, both in the relationships within the echeneidae and the sister-group relationship, from the morphology-based hypothesis of O’Toole (2002) and the mtDNA-based hypothesis of Gray et al. (2009). Our date estimates are comparable to older ages hypothesized in Harrington et al. (2016). However, our study is the first fossil-calibrated phylogeny that includes all echeneid taxa, making our dates difficult to compare with other studies.

Although molecular phylogenetic studies based solely on datasets comprising only a few loci have provided valuable insights into the relationships of innumerable groups of fishes, the sparse genomic coverage of these datasets leaves such analysis susceptible to the degradation of phylogenetic signals due to incomplete lineage sorting, among other factors. This, in turn, has the potential to mislead comparative analysis and render their results erroneous. The resolution of echeneid relationships through UCE analysis has produced a reliable framework on which our comparative analysis is based. We hope this study demonstrates that historically difficult phylogenetic questions can be addressed with phylogenomic approaches and that the integration of phylogenomic inference can strengthen the power of comparative research.

Like Friedman et al. (2013), our analysis provides strong support for the hypothesis that a clade comprised of *Coryphaena* and *Rachycentron* is the sister group of the Echeneidae. This hypothesis is also congruent with Gray et al. (2009) and larval evidence published by Johnson (1984). This challenges O’Toole (2002), who hypothesized that *Rachycentron* is the sister taxon of the remoras to the exclusion of *Coryphaena*.

### Echeneid host diversity

Our results challenge the reef-generalists, pelagic-specialist framework previously proposed by Cressey and Lachner (1970) and O’Toole (2002). Unlike these previous studies, which took into account only the number of host species, our analysis assessed the MPD among host for each species. In so doing, we found that one of the reef-generalists species, *E. naucrates*, demonstrates significant host specialization. Despite associations with a rather large number of hosts (Supplementary Table S7), *E. naucrates* has avoided several large clades of marine vertebrates, including lamniform sharks, scombrids, billfishes, and cetaceans (Fig. 4). By skipping these clades in its host diversification, MPD for *E. naucrates* is reduced and the analysis reveals phylogenetic unevenness. Similarly, within the pelagic-specialist clade, we found that only two species, *R. osteochir*, the marlin sucker, and *R. australis*, the whalesucker, had significant phylogenetic clustering. All other species within the pelagic clade had MPD SES values close to 0.

We note that our host-association data include substantially different numbers of observations between species, ranging from as few as nine observations for *P. lineatus* to hundreds for *R. osteochir*. With additional host-association observations of relatively rare species like. *P. lineatus*, host specificity as determined by MPD SES analysis should be reevaluated.

### Disc morphology and host characteristics

A disc morphospace based on the first two PCs, accounting for a total of 70% of the morphological variance, does not distinguish the reef and pelagic clades. Thus, anatomical diversification of the remora disc system cannot be explained simply in terms of habitat, that is, reef versus pelagic. We did, however, uncover important relationships between major axes of disc morphospace and host characteristics that may be crucial in effective adhesion. We found significant relationships in our models between PC1 and PC2 and the best-fitting independent variables for these components (*S*_a_ and both MPD obs. and Re, respectively); however, we did not find a significant relationship in the best-fitting model for PC3. We therefore restrict our discussion to PC1 and PC2.

Principle component 1, which explains 45% of disc morphospace variance was best explained by a simple model that accounts for only host *S*_a_, feature or asperity height (Table 2). PC1 loads heavily for shorter medial spinule lengths, shorter lamella processes, wider intercalary cups, shorter intercalary process lengths, narrower heads of the interneural rays, shorter interneural rays, and longer spinule lengths. This indicates the importance of these features in adhesion to surfaces of variable roughness. The lamella processes serve as the point of insertion for the fin erector muscles (Fulcher and Motta 2006) which we propose pull on the lamella at its process, which in turn, rotates the lamella dorsocranially about its articulations with the dorsal head of interneural ray and the broadened posterior face of the intercalary process (Fig. 2). In this configuration, the lamella process acts as an inlever, imparting muscular force through the lamella against the host surface, decreasing intra-disc pressure. The actions of longer lamella processes would result in greater magnitudes of subambient pressure, collapsing the soft tissue along the margin of the disc against the profile of the host surface forming a strong seal, while shorter processes would result in lesser magnitudes of subambient pressure and a weaker seal. In general, those species with shorter processes adhere to smoother hosts, indicating that disc suction may be more important for adhesion in these species. Using a biorobotic model of the disc system of *E. naucrates*, Wang et al. (2017) demonstrated that these mechanical linkages could impart considerable forces against mako shark skin—a rather smooth surface (Supplementary Table S4)—and an effective seal along a soft disc perimeter.

The positive correlation of wider intercalary cups and negative correlation of intercalary process length with host *S*_a_ may be explained by the potential role of the intercalary bones as elements against which the lamellae rotate as described above. The ventral surface of the lamella articulates with the intercalary cup. A broader cup would provide more support for the lamella and rigidify the lamella–intercalary system as it interacts with rougher surfaces. A stouter intercalary process would deform less under the stress of lamella rotation and more effectively facilitate the generation force against the host surface by the spinules of the lamella. Both features, in turn, may increase magnitudes of disc subambient pressure, adhesion force, and pull-off resistance on rough surfaces.

For PC1, the combination of positive coefficients for spinule density and negative coefficients for spinule and medial spinule lengths suggests that many smaller structures play an important role in interacting with rough host profiles. Through experimental (Fulcher and Motta 2006), computational (Beckert et al. 2015), and biorobotic (Wang et al. 2017) approaches, others have found that spinules increase friction and shear resistance in *E. naucrates*. In finding a positive relationship between spinule density and host surface roughness, our comparative analysis corroborates this hypothesis derived from these more narrowly focused studies, extending it across the group. Contrary to our results, smaller, compliant micro-scale spines have been found to be more effective in adhesion to smoother surfaces for insects (Bullock and Federle 2011) and insect-inspired biomimetic systems (Asbeck et al. 2006). This raises the possibility that remora spinule length is tuned to some other measure of roughness not measured in this study that covaries with asperity height (*S*_a_, e.g., spacing of asperities).

The contribution of shorter interneural lengths and narrower heads of the interneural rays to PC1 indicates that a smaller interneural bone is associated with effective attachment to rough surfaces or, conversely, more robust interneurals are associated with smooth host systems. Each lamella rotates in the transverse plane about the lateral edge of the broadened head of the interneural ray (Britz and Johnson 2012) which is embedded in the axial musculature. We interpret the more robust interneural rays as an anatomical specialization that stabilizes these linkages. A more rigid lamella system anchored by robust interneurals would permit more effective transmission of force against the host surface. Although a suction-based system does not effectively resist shear, this may cause the spinules to embed into the host surface—especially a compliant one—during engagement of the disc and therefore increase shear resistance. The feasibility of such a scenario should be evaluated in the context of host surface compliance in future work.

Principle component 2, which captures 25% of disc morphospace variance, was best explained by two simple models with similar levels of wAICc support, one explained by MPD obs. and the other by lower host maximum Reynolds number (Re; Table 2). PC2 loads heavily and negatively for disc length and lamella count and positively for intercalary bone and lamella aspect ratios. Thus, larger and wider discs with fewer and more expansive lamella systems (in the rostrocaudal axis) are associated with higher Re and increased drag forces. As Re increases, the drag coefficient decreases for streamlined bodies (Munson et al. 2006). However, for species attached to larger, faster hosts, this results in higher flow velocities and greater drag forces exerted on the body. Using computational fluid experiments, Beckert et al. (2016) found that *E. naucrates* experiences increased drag forces with increasing Re. We assert that larger disc systems generate more suction and shear resistance to counter increased drag.

In addition, the positive relationship between PC2 and MPD obs. values indicates that larger disc size and lamella systems were important in the evolution of host phylogenetic specialization. That PC2 was also explained by a model incorporating host Re suggests that host phylogenetic distance (MPD obs.) covaries with Re. This may be explained by an overall trend of specialists preferring clades of typically larger species. For example, the specialists *R. australis* and *R. osteochir* prefer cetaceans and billfishes, respectively, clades of some of the largest aquatic vertebrates. These larger taxa attain higher maximum velocities and, in turn, higher maximum Re.

Lastly, our comparative analyses offer key insights into the adhesion of the echeneids to a wide variety of hosts. This work focuses on the relationship between the osteology of the suction-disc system and a limited set of host variables. We note that more extensive analysis of disc soft tissues (e.g., skin and musculature) and other bony elements (e.g., spinule shape, e.g., Beckert et al. 2015) may more fully elucidate the biomechanical basis of adhesion in the Echeneidae.

In addition, we hope that this and future studies of the remora disc system will serve as the basis for deeper understanding of biological adhesion and impact the expanding fields of biorobotics and biomimetics. Specifically, we hope that engineers interested in artificial adhesion devices that perform well over variably rough surfaces will continue to use the results of comparative research to inform device design (Gorb 2008; Wang et al. 2017).

## Conclusion

Our UCE-based phylogenetic hypothesis, the first phylogenomic analysis for the group, supports a monophyletic Echeneidae, monophyletic pelagic, and reef clades, and a sister relationship between the echeneids and a clade consisting of *Rachycentron* and *Coryphaena*. This extremely well-supported topology for the echeneidae was used in phylogenetic PCA to establish a disc phylomorphospace. We found that variation in disc morphospace was dispersed and that pelagic-reef distinction was only represented in a morphospace that considered PC3 which accounted for only 16% of disc morphological variation. In addition, the specificity of host choice as determined by MPD is more variable in the pelagic-specialist group and less variable in the reef-generalist group than previously proposed, a result that challenges these host-specificity classifications. Through ordinary linear models of phylogenetic PCA components and simple model choice operations, we found that the major axes of disc morphospace—the first and second PCs—are best explained by models that include host skin roughness (PC1) and host MPD and maximum swimming Re (both PC2). Integrating these results, we conclude that ecological and morphological diversification was driven by the selection pressures of host skin surface roughness, and specialization to host size and hydrodynamic regime.

## Author contributions

C.P.K. conceived the study, performed all comparative analysis, and wrote the manuscript. A.S. helped design the study and write the manuscript, undertook all morphological analysis, and assembled host surface data. W.L.L. and P.C. helped write the manuscript, assembled UCE data, and performed phylogenetic analysis. All authors gave final approval for publication.

## Supplementary Material

Supplementary_Table_obz007
