# Supplementary material for: Comparative Functional and Phylogenomic Analyses of Host Association in the Remoras (Echeneidae), a Family of Hitchhiking Fishes
Source: Integr Org Biol. 2019 May 10;1(1):obz007. doi: 10.1093/iob/obz007 (PMC7671162; doi:10.1093/iob/obz007)
Supplement: Supplementary_Table_obz007 [file supplementary_table_obz007.zip › Tables1.docx]

Table s1: Host associations for the eight species of the family Echeneidae. Data are compiled from O’Toole (2002) and from querying natural-history museum records through the Global Biodiversity Information Facility (GBIF) database. “x” in the GBIF column indicates the host includes a taxon record from GBIF

GBIF

| host | E. naucrates | E. neucratoides | P. lineatus | R. australis | R. brachyptera | R. osteochir | R. remora | R. albescens |  | Family | Group |
| --- | --- | --- | --- | --- | --- | --- | --- | --- | --- | --- | --- |
| *Alopias superciliosus* | 0 | 0 | 0 | 0 | 0 | 0 | 1 | 0 |  | Alopiidae | Sharks |
| *Carcharias* sp. | 1 | 0 | 0 | 0 | 0 | 0 | 7 | 0 |  | Odontaspididae | Sharks |
| *Carcharias taurus* | 1 | 0 | 0 | 0 | 0 | 0 | 0 | 0 | x | Odontaspididae | Sharks |
| *Carcharhinus falciformis* | 3 | 3 | 0 | 0 | 1 | 0 | 54 | 0 |  | Carcharhinidae | Sharks |
| *Carcharhinus leucas* | 1 | 0 | 0 | 0 | 0 | 0 | 0 | 0 |  | Carcharhinidae | Sharks |
| *Carcharhinus limbatus* | 2 | 0 | 0 | 0 | 0 | 0 | 0 | 0 |  | Carcharhinidae | Sharks |
| *Carcharhinus longmimanus* | 0 | 0 | 0 | 0 | 0 | 0 | 131 | 0 |  | Carcharhinidae | Sharks |
| *Carcharhinus melanopterus* | 0 | 0 | 0 | 0 | 0 | 0 | 1 | 0 |  | Carcharhinidae | Sharks |
| *Carcharhinus melanopterus* | 1 | 0 | 0 | 0 | 0 | 0 | 0 | 0 | x | Carcharhinidae | Sharks |
| *Carcharhinus obscurus* | 0 | 2 | 1 | 0 | 0 | 0 | 5 | 0 |  | Carcharhinidae | Sharks |
| *Carcharhinus* sp. | 1 | 0 | 0 | 0 | 0 | 0 | 7 | 0 |  | Carcharhinidae | Sharks |
| *Galeocerdo cuvieri* | 1 | 0 | 0 | 0 | 0 | 0 | 9 | 1 |  | Carcharhinidae | Sharks |
| *Ginglymostoma cirratum* | 3 | 0 | 0 | 0 | 0 | 0 | 0 | 0 |  | Ginglymostomatidae | Sharks |
| *Isurus oxyrinchus* | 0 | 0 | 0 | 0 | 1 | 1 | 23 | 0 |  | Lamnidae | Sharks |
| *Isurus paucus* | 0 | 0 | 0 | 0 | 3 | 0 | 8 | 0 |  | Lamnidae | Sharks |
| *Isurus* sp. | 0 | 0 | 0 | 0 | 0 | 0 | 1 | 0 |  | Lamnidae | Sharks |
| *Prionace glauca* | 0 | 0 | 0 | 0 | 187 | 0 | 551 | 0 |  | Carcharhinidae | Sharks |
| *Pseudocarcharias kamoharai* | 0 | 0 | 1 | 0 | 0 | 0 | 0 | 0 |  | Pseudocarchariidae | Sharks |
| *Negaprion brevirostris* | 6 | 0 | 0 | 0 | 0 | 0 | 0 | 0 |  | Carcharhinidae | Sharks |
| *Rhincodon typus* | 0 | 0 | 0 | 0 | 0 | 0 | 4 | 0 |  | Rhincodontidae | Sharks |
| *Rhizoprionodon acutus* | 1 | 0 | 0 | 0 | 0 | 0 | 1 | 0 |  | Carcharhinidae | Sharks |
| *Sphyrna zygaena* | 0 | 0 | 0 | 0 | 2 | 0 | 34 | 0 |  | Sphyrnidae | Sharks |
| *Triaenodon obesus* | 1 | 0 | 0 | 0 | 0 | 0 | 0 | 0 |  | Carcharhinidae | Sharks |
| Unidentified sharks | 5 | 0 | 1 | 0 | 3 | 0 | 34 | 2 |  |  | Sharks |
| *Mobula birostris* | 0 | 0 | 0 | 0 | 1 | 0 | 3 | 27 |  | Myliobatidae | Batoids |
| *Myliobatidae* sp. | 3 | 0 | 0 | 0 | 0 | 0 | 0 | 0 |  | Myliobatidae | Batoids |
| *Mobula lucasana* | 0 | 0 | 1 | 0 | 0 | 0 | 0 | 1 | x | Myliobatidae | Batoids |
| *Caretta caretta* | 0 | 0 | 1 | 0 | 0 | 0 | 0 | 0 | x | Cheloniidae | Chelonians |
| Turtles | 2 | 0 | 3 | 0 | 0 | 0 | 10 | 0 |  | Cheloniidae | Chelonians |
| *Delphinus capensis* | 0 | 0 | 0 | 6 | 0 | 0 | 0 | 0 |  | Delphinidae | Odontocetes |
| *Delphinus delphis* | 0 | 0 | 0 | 7 | 0 | 0 | 0 | 0 |  | Delphinidae | Odontocetes |
| *Stenella plagiodon* | 0 | 0 | 0 | 1 | 0 | 0 | 0 | 0 |  | Delphinidae | Odontocetes |

| *Dolphin* sp. | 0 | 0 | 0 | 2 | 0 | 0 | 0 | 0 |  | Delphinidae | Odontocetes |
| --- | --- | --- | --- | --- | --- | --- | --- | --- | --- | --- | --- |
| *Physeter macrocephalus* | 0 | 0 | 0 | 2 | 0 | 0 | 0 | 0 |  | Physeteridae | Odontocetes |
| *Balaenoptera musculus* | 0 | 0 | 0 | 19 | 0 | 0 | 0 | 0 |  | Balaenopteridae | Mysticetes |
| Cetaceans | 2 | 2 | 0 | 6 | 0 | 0 | 0 | 0 | x |  | Mysticetes |
| *Megalops atlanticum* | 5 | 0 | 0 | 0 | 0 | 0 | 0 | 0 | x | Megalopidae | Actinops |
| *Gadidae* sp. | 0 | 0 | 0 | 0 | 0 | 0 | 1 | 0 |  | Gadidae | Actinops |
| Epinephelini sp. | 1 | 0 | 0 | 0 | 0 | 0 | 0 | 0 |  | Serranidae | Actinops |
| *Epinephelus itajara* | 1 | 0 | 1 | 0 | 0 | 0 | 0 | 0 |  | Serranidae | Actinops |
| *Lutjanus* sp. | 1 | 0 | 0 | 0 | 0 | 0 | 0 | 0 |  | Lutjanidae | Actinops |
| *Lutjanus apodus* | 1 | 0 | 0 | 0 | 0 | 0 | 0 | 0 | x | Lutjanidae | Actinops |
| *Diplodus holbrooki* | 1 | 0 | 0 | 0 | 0 | 0 | 0 | 0 |  | Sparidae | Actinops |
| *Ostracion* sp. | 1 | 0 | 0 | 0 | 0 | 0 | 0 | 0 |  | Ostraciidae | Actinops |
| *Masturus lanceolatus* | 0 | 0 | 0 | 0 | 2 | 0 | 1 | 0 |  | Molidae | Actinops |
| *Masturus lanceolatus* | 0 | 0 | 0 | 0 | 2 | 0 | 0 | 0 | x | Molidae | Actinops |
| *Mola mola* | 0 | 0 | 0 | 0 | 3 | 1 | 0 | 0 |  | Molidae | Actinops |
| *Lactophrys polygonia* | 1 | 0 | 0 | 0 | 0 | 0 | 0 | 0 |  | Ostraciidae | Actinops |
| *Lactophrys quadricornis* | 1 | 0 | 0 | 0 | 0 | 0 | 0 | 0 |  | Ostraciidae | Actinops |
| *Lactophrys triqueter* | 2 | 0 | 0 | 0 | 0 | 0 | 0 | 0 |  | Ostraciidae | Actinops |
| *Diodon* sp. | 0 | 0 | 3 | 0 | 0 | 0 | 0 | 0 |  | Diodontidae | Actinops |
| *Scarus guacamaia* | 1 | 0 | 0 | 0 | 0 | 0 | 0 | 0 |  | Scaridae | Actinops |
| *Scarus taeniopterus* | 1 | 0 | 0 | 0 | 0 | 0 | 0 | 0 |  | Scaridae | Actinops |
| *Sparisoma aurofrenatum* | 2 | 0 | 0 | 0 | 0 | 0 | 0 | 0 |  | Scaridae | Actinops |
| *Sparisoma chrysopterum* | 2 | 0 | 0 | 0 | 0 | 0 | 0 | 0 |  | Scaridae | Actinops |
| *Cheilinus undulatus* | 1 | 0 | 0 | 0 | 0 | 0 | 0 | 0 | x | Labridae | Actinops |
| *Cirrhilabrus rubripinnis* | 2 | 0 | 0 | 0 | 0 | 0 | 0 | 0 |  | Labridae | Actinops |
| *Sparisoma viride* | 1 | 0 | 0 | 0 | 0 | 0 | 0 | 0 |  | Scaridae | Actinops |
| Scaridae sp. | 2 | 0 | 0 | 0 | 0 | 0 | 0 | 0 |  | Scaridae | Actinops |
| Sparidae sp. | 1 | 0 | 0 | 0 | 0 | 0 | 0 | 0 | x | Scaridae | Actinops |
| Serranidae sp. | 1 | 0 | 0 | 0 | 0 | 0 | 0 | 0 |  | Serranidae | Actinops |
| *Sphyraena barracuda* | 2 | 0 | 2 | 0 | 0 | 0 | 0 | 0 |  | Sphyraenidae | Actinops |
| Sphyraena sp. | 7 | 0 | 2 | 0 | 1 | 0 | 0 | 0 |  | Sphyraenidae | Actinops |
| Tetraodontidae sp. | 0 | 0 | 1 | 0 | 0 | 0 | 0 | 0 |  | Tetraodontidae | Actinops |
| *Trachinotus* sp. | 1 | 0 | 0 | 0 | 0 | 0 | 0 | 0 |  | Carangidae | Actinops |
| *Acanthocybium solandri* | 0 | 0 | 0 | 0 | 0 | 1 | 0 | 0 |  | Scomridae | Actinops |
| *Thunnus albacares* | 0 | 0 | 0 | 0 | 0 | 0 | 1 | 0 |  | Scombridae | Actinops |
| *Thunnus obesus* | 0 | 0 | 0 | 0 | 0 | 0 | 13 | 0 |  | Scombridae | Actinops |
| *Istiophorus albicans* | 0 | 0 | 0 | 0 | 32 | 112 | 0 | 0 |  | Istiophoridae | Billfishes + Swordfish |
| *Istiophorus greyi* | 0 | 0 | 0 | 0 | 0 | 0 | 1 | 0 |  | Istiophoridae | Billfishes + Swordfish |
| *Istiophorus* sp. | 0 | 0 | 0 | 0 | 0 | 11 | 1 | 0 |  | Istiophoridae | Billfishes + Swordfish |

| Istiophorinae sp. | 0 | 0 | 0 | 0 | 17 | 20 | 0 | 0 |  | Istiophoridae | Billfishes + Swordfish |
| --- | --- | --- | --- | --- | --- | --- | --- | --- | --- | --- | --- |
| *Makaira indica* | 0 | 0 | 0 | 0 | 28 | 106 | 3 | 0 |  | Istiophoridae | Billfishes + Swordfish |
| *Makaira nigricans* | 0 | 0 | 0 | 0 | 8 | 35 | 0 | 0 |  | Istiophoridae | Billfishes + Swordfish |
| *Kajikia albida* | 0 | 0 | 0 | 0 | 12 | 94 | 13 | 0 |  | Istiophoridae | Billfishes + Swordfish |
| *Tetrapturus angustirostris* | 0 | 0 | 0 | 0 | 0 | 5 | 0 | 0 |  | Istiophoridae | Billfishes + Swordfish |
| *Kajikia audax* | 0 | 0 | 0 | 0 | 39 | 89 | 1 | 0 |  | Istiophoridae | Billfishes + Swordfish |
| *Tetrapturus belone* | 0 | 0 | 0 | 0 | 3 | 6 | 0 | 0 |  | Istiophoridae | Billfishes + Swordfish |
| *Tetrapturus pfluegeris* | 0 | 0 | 0 | 0 | 0 | 2 | 0 | 0 |  | Istiophoridae | Billfishes + Swordfish |
| *Tetrapturus* sp. | 0 | 0 | 0 | 0 | 0 | 3 | 0 | 0 |  | Istiophoridae | Billfishes + Swordfish |
| *Xiphias gladius* | 0 | 1 | 0 | 0 | 275 | 5 | 2 | 0 |  | Xiphiidae | Billfishes + Swordfish |
| Unidentified billfish | 0 | 0 | 0 | 0 | 0 | 1 | 0 | 0 |  |  | Billfishes + Swordfish |
| *Seriola dumerili* | 1 | 0 | 0 | 0 | 0 | 0 | 0 | 0 |  | Carangidae | Actinops |
| *Caranx ruber* | 2 | 0 | 0 | 0 | 0 | 0 | 0 | 0 |  | Carangidae | Actinops |
| *Caranx hippos* | 1 | 0 | 0 | 0 | 0 | 0 | 0 | 0 | x | Carangidae | Actinops |
| *Coryphaenidae* sp. | 0 | 0 | 0 | 0 | 0 | 2 | 0 | 0 |  | Coryphaenidae | Actinops |
| *Platax teira* | 1 | 0 | 0 | 0 | 0 | 0 | 0 | 0 | x | Ephippidae | Actinops |
