# Supplementary material for: Comparative Functional and Phylogenomic Analyses of Host Association in the Remoras (Echeneidae), a Family of Hitchhiking Fishes
Source: Integr Org Biol. 2019 May 10;1(1):obz007. doi: 10.1093/iob/obz007 (PMC7671162; doi:10.1093/iob/obz007)
Supplement: Supplementary_Table_obz007 [file supplementary_table_obz007.zip › Tables2.docx]

Table s2: GenBank Accession numbers of five gene fragments used in phylogenetic reconstruction of vertebrate hosts of the Echeneidae. Column values containing “Ex.” indicate the exemplar used for that gene fragment when host species was not found in GenBank

| host | COI Ex. | COI GB#. | RAG Ex. | RAG GB# | Rho Ex. | Rho GB# ENC Ex. ENC GB# IRBP Ex IRBP GB# |
| --- | --- | --- | --- | --- | --- | --- |
| *Petromyzon marinus* | *Petromyzon*  *marinus* | KX145569.1 |  |  | *Petromyzon*  *marinus* | AH005459.2 |
| *Alopias superciliosus* | *Alopias super-*  *ciliosus* | EU400162.1 | *Alopias super-*  *ciliosus* | AF135481.1 |  |  |
| *Carcharias* sp. | *Carcharias*  *taurus* | JF493075.1 | *Carcharias*  *taurus* | AF135475.1 |  |  |
| *Carcharias taurus* | *Carcharias*  *taurus* | JF493075.1 | *Carcharias*  *taurus* | AF135475.1 |  |  |
| *Carcharhinus falciformis* | *Carcharhinus*  *falciformis* | EU398613.1 | *Carcharhinus*  *falciformis* | JX978319.1 |  |  |
| *Carcharhinus leucas* | *Carcharhinus*  *leucas* | EU818710.1 |  |  |  |  |
| *Carcharhinus limbatus* | *Carcharhinus*  *limbatus* | KU366625.1 |  |  |  |  |
| *Carcharhinus melanopterus* | *Carcharhinus*  *melanopterus* | JN082190.1 |  |  |  |  |
| *Carcharhinus melanopterus* | *Carcharhinus*  *melanopterus* | JN082190.1 |  |  |  |  |
| *Carcharhinus obscurus* | *Carcharhinus*  *obscurus* | JF493066.1 |  |  |  |  |
| *Carcharhinus* sp. | *Carcharhinus*  *tilstoni* | KU366636.1 | *Carcharhinus*  *falciformis* | JX978319.1 |  |  |
| *Galeocerdo cuvieri* | *Galeocerdo cu-*  *vier* | JF493501.1 |  |  |  |  |
| *Ginglymostoma cirratum* | *Nebrius*  *ferrugineus* | KP193447.1 |  |  | *Rhincodon ty-*  *pus* | XM020522486.1 |
| *Isurus oxyrinchus* | *Isurus*  *oxyrinchus* | KU366629.1 | *Isurus*  *oxyrinchus* | AF135480.1 |  |  |
| *Isurus paucus* | *Isurus paucus* | EU398900.1 |  |  |  |  |
| *Isurus* sp. | *Isurus*  *oxyrinchus* | KU366629.1 | *Isurus*  *oxyrinchus* | AF135480.1 |  |  |
| *Prionace glauca* | *Prionace*  *glauca* | KY176584.1 |  |  |  |  |

36

| *Pseudocarcharias kamoharai* | *Pseudocarcharias*  *kamoharai* | EU398990.1 | *Pseudocarcharias*  *kamoharai* | AF135479.1 |  | | | | | |
| --- | --- | --- | --- | --- | --- | --- | --- | --- | --- | --- |
| *Negaprion brevirostris* | *Negaprion*  *brevirostris* | FJ519631.1 |  |  |  |  |  |  |  |  |
| *Rhincodon typus* | *Rhincodon ty-*  *pus* | EU398993.1 | *Ginglymostoma*  *cirratum* | U13982.1 | *Rhincodon ty-*  *pus* | XM020522486.1 |  |  |  |  |
| *Rhizoprionodon acutus* | *Rhizoprionodon*  *acutus* | FJ519253.1 | *Rhizoprionodon*  *oligolinx* | JX978318.1 |  |  |  |  |  |  |
| *Sphyrna zygaena* | *Sphyrna*  *zygaena* | JF494565.1 | *Sphyrna*  *lewini* | JX978315.1 | *Sphyraena*  *sphyraena* | AY141312.1 |  |  |  |  |
| *Triaenodon obesus* | *Triaenodon*  *obesus* | FJ519289.1 | *Carcharhinus*  *falciformis* | JX978319.1 |  |  |  |  |  |  |
| *Istiophorus* sp. | *Istiophorus*  *platypterus* | KU945067.1 |  |  | *Istiophorus*  *platypterus* | DQ874808.1 |  |  | *Istiophorus*  *platypterus* | JQ939150.1 |
| Istiophorinae sp. | *Kajikia audax* | KX781884.1 |  |  | *Istiophorus*  *platypterus* | DQ874808.1 | *Tetrapturus*  *angustirostris* | KF139605.1 | *Istiophorus*  *platypterus* | JQ939150.1 |
| *Makaira indica* | *Istiompax in-*  *dica* | KU945066.1 |  |  | *Makaira*  *nigricans* | DQ874810.1 |  |  |  |  |
| *Makaira nigricans* | *Makaira*  *nigricans* | HQ945883.1 |  |  | *Makaira*  *nigricans* | DQ874810.1 | *Tetrapturus*  *angustirostris* | KF139605.1 |  |  |
| *Kajikia albida* | *Kajikia albida* | HM909826.1 |  |  |  |  |  |  |  |  |
| *Tetrapturus angustirostris* | *Tetrapturus*  *angustirostris* | EU489702.1 |  |  | *Tetrapturus*  *angustirostris* | DQ080346.1 | *Tetrapturus*  *angustirostris* | KF139605.1 |  |  |
| *Kajikia audax* | *Kajikia audax* | KX781884.1 |  |  | *Kajikia audax* | HQ630754.1 |  |  |  |  |
| *Tetrapturus belone* | *Tetrapturus*  *belone* | HQ024833.1 |  |  |  |  |  |  |  |  |
| *Tetrapturus* sp. | *Tetrapturus*  *angustirostris* | EU489702.1 |  |  | *Tetrapturus*  *angustirostris* | DQ080346.1 | *Tetrapturus*  *angustirostris* | KF139605.1 | *Istiophorus*  *platypterus* | JQ939150.1 |
| *Xiphias gladius* | *Xiphias glad-*  *ius* | KX781925.1 |  |  | *Xiphias glad-*  *ius* | EU638019.1 | *Xiphias glad-*  *ius* | KF139613.1 | *Xiphias glad-*  *ius* | EU638163.1 |
| Sting ray | *Rhinoptera*  *marginata* | KX688095.1 | *Aetomylaeus*  *bovinus* | KX485398.1 |  |  |  |  |  |  |
| *Mobula lucasana* | *Mobula tara-*  *pacana* | KY454873.1 | *Mobula*  *thurstoni* | KX485393.1 |  |  |  |  |  |  |
| *Delphinus delphis* | *Delphinus*  *delphis* | EU139278.1 |  |  | *Delphinus*  *delphis* | AF055314.1 |  |  | *Delphinus*  *delphis* | AF304077.1 |
| *Stenella plagiodon* | *Stenella*  *frontalis* | EF090646.1 | *Lagenorhynchus*  *obscurus* | AY239179.1 |  |  |  |  | *Stenella*  *coeruleoalba* | KM101414.1 |

37

| *Balaenoptera musculus* | *Balaenoptera*  *omurai* | KP230448.1 | *Balaenoptera*  *physalus* | AY239180.1 | *Balaenoptera*  *acutorostrata* | XM007192608.1 |  |  | *Balaenoptera*  *acutorostrata* | U50820.1 |
| --- | --- | --- | --- | --- | --- | --- | --- | --- | --- | --- |
| Cetaceans | *Balaenoptera*  *omurai* | KP230448.1 |  |  |  |  |  |  |  |  |
| *Caretta caretta* | *Caretta caretta* | GQ152889.1 | *Caretta caretta* | FJ009032.1 | *Chelonia my-*  *das* | XM007059947.1 |  |  |  |  |
| Epinephelini sp. | *Cephalopholis*  *hemistiktos* | KY676116.1 | *Epinephelus*  *lanceolatus* | KP739750.1 | *Epinephelus*  *fasciatus* | JX093970.1 | *Paranthias*  *colonus* | HM050009.1 | *Epinephelus*  *aeneus* | DQ168064.1 |
| *Epinephelus itajara* | *Epinephelus*  *itajara* | JN021300.1 | *Epinephelus*  *lanceolatus* | KP739750.1 | *Epinephelus*  *itajara* | DQ197854.1 | *Epinephelus*  *aeneus* | HM049986.1 | *Epinephelus*  *aeneus* | DQ168064.1 |
| *Lactophrys polygonia* | *Acanthostracion*  *polygonius* | JQ861011.1 |  |  | *Acanthostracion*  *polygonius* | JQ861035.1 | *Ostracion cu-*  *bicus* | KF027638.1 | *Acanthostracion*  *polygonius* | JQ861128.1 |
| *Lactophrys quadricornis* | *Acanthostracion*  *quadricornis* | GU225088.1 |  |  | *Lactophrys*  *trigonus* | JQ861041.1 | *Acanthostracion*  *quadricornis* | KF027636.1 | *Acanthostracion*  *quadricornis* | KF027719.1 |
| *Lactophrys triqueter* | *Rhinesomus*  *triqueter* | KF930357.1 |  |  | *Lactophrys*  *triqueter* | JQ861042.1 | *Rhinesomus*  *triqueter* | JX189007.1 | *Rhinesomus*  *triqueter* | JQ861135.1 |
| *Lutjanus* sp. | *Lutjanus fulv-*  *iflamma* | KY675445.1 |  |  | *Lutjanus*  *sebae* | EU637974.1 | *Lutjanus*  *griseus* | KF139514.1 | *Lutjanus*  *sebae* | EU638134.1 |
| *Lutjanus apodus* | *Lutjanus apo-*  *dus* | KX223918.1 |  |  |  |  |  |  |  |  |
| *Megalops atlanticus* | *Megalops*  *cyprinoides* | KU942719.1 |  |  | *Megalops*  *cyprinoides* | KY026026.1 |  |  |  |  |
| *Megalops atlanticum* | *Megalops*  *cyprinoides* | KU942719.1 |  |  | *Megalops*  *cyprinoides* | KY026026.1 |  |  |  |  |
| *Masturus lanceolatus* | *Masturus*  *lanceolatus* | KU945271.1 |  |  | *Mola mola* | AF137215.1 | *Masturus*  *lanceolatus* | EF539263.1 | *Mola mola* | DQ168087.1 |
| *Scarus taeniopterus* | *Scarus tae-*  *niopterus* | JQ843040.1 |  |  |  |  | *Scarus globi-*  *ceps* | KF139565.1 | *Scarus hoefleri* | DQ168112.1 |
| *Sparisoma aurofrenatum* | *Sparisoma au-*  *rofrenatum* | GU224590.1 |  |  |  |  |  |  |  |  |
| *Sparisoma chrysopterum* | *Sparisoma*  *chrysopterum* | GU225041.1 |  |  |  |  |  |  |  |  |
| *Cheilinus undulatus* | *Cheilinus un-*  *dulatus* | KY676079.1 |  |  |  |  |  |  |  |  |
| *Cirrhilabrus rubripinnis* | *Cirrhilabrus*  *lineatus* | KX037920.1 | *Semicossyphus*  *pulcher* | FJ616726.1 | *Cirrhilabrus*  *rubripinnis* | KP881287.1 |  |  | *Xyrichtys no-*  *vacula* | EU638164.1 |
| *Sparisoma viride* | *Sparisoma*  *viride* | GU225053.1 | *Semicossyphus*  *pulcher* | FJ616726.1 |  |  |  |  | *Xyrichtys no-*  *vacula* | EU638164.1 |

| Scaridae sp. | *Chlorurus*  *sordidus* | KY676194.1 |  |  |  |  |  |  |  |  |
| --- | --- | --- | --- | --- | --- | --- | --- | --- | --- | --- |
| Sparidae |  |  |  |  |  |  | *Pagrus pagrus* | KF139558.1 |  |  |
| *Diplodus holbrooki* | *Diplodus hol-* | KJ012344.1 |  |  | *Diplodus vul-* | Y18663.1 |  |  |  |  |
|  | *brookii* |  |  |  | *garis* |  |  |  |  |  |
| Serranidae | *Cephalopholis* | KY676116.1 |  |  |  |  | *Grammistes* | KF139554.1 | *Serranus* | DQ168115.1 |
|  | *hemistiktos* |  |  |  |  |  | *sexlineatus* |  | *accraensis* |  |
| *Thunnus albacares* | *Thunnus* | KY656483.1 |  |  | *Thunnus* | EF427525.1 | *Thunnus* | KF139609.1 |  |  |
|  | *albacares* |  |  |  | *albacares* |  | *albacares* |  |  |  |
| *Thunnus obesus* | *Thunnus obe-* | KY656482.1 |  |  | *Thunnus obe-* | DQ197915.1 | *Thunnus* | KF139609.1 | *Scomber* | DQ168113.1 |
|  | *sus* |  |  |  | *sus* |  | *albacares* |  | *scombrus* |  |
| *Sphyraena barracuda* | *Sphyraena* | KX119510.1 |  |  | *Sphyraena* | DQ874816.1 | *Sphyraena* | KF139610.1 | *Sphyraena* | JQ939166.1 |
|  | *barracuda* |  |  |  | *barracuda* |  | *barracuda* |  | *barracuda* |  |
| *Sphyraena* sp. | *Sphyraena qe-* | KY676078.1 |  |  | *Sphyraena* | AY141312.1 | *Sphyraena* | KF139611.1 | *Sphyraena* | DQ168118.1 |
|  | *nie* |  |  |  | *sphyraena* |  | *japonica* |  | *sphyraena* |  |
| Tetraodontidae sp. |  |  |  |  | *Takifugu* | NM001033849.1 | *Sphoeroides* | EF539264.1 | *Tetraodon* | EU625595.1 |
|  |  |  |  |  | *rubripes* |  | *dorsalis* |  | *mbu* |  |
| *Trachinotus* sp. | *Trachinotus* | KY176674.1 |  |  | *Trachinotus* | AY141314.1 | *Trachinotus* | KF139479.1 | *Trachinotus* | JQ939168.1 |
|  | *ovatus* |  |  |  | *ovatus* |  | *ovatus* |  | *falcatus* |  |
| *Acanthocybium solandri* | *Acanthocybium* | KU945040.1 |  |  | *Acanthocybium* | DQ874804.1 | *Thunnus* | KF139609.1 |  |  |
|  | *solandri* |  |  |  | *solandri* |  | *albacares* |  |  |  |
| *Caranx ruber* | *Carangoides* | MF041726.1 |  |  | *Caranx sex-* | JQ938003.1 | *Caranx crysos* | KF139476.1 | *Caranx sex-* | JQ939161.1 |
|  | *ruber* |  |  |  | *fasciatus* |  |  |  | *fasciatus* |  |
| *Caranx hippos* | *Caranx hippos* | JN021293.1 |  |  |  |  |  |  |  |  |
| *Coryphaena* sp. | *Coryphaena* | KX781874.1 |  |  | *Coryphaena* | EU637951.1 | *Coryphaena* | KF139497.1 | *Coryphaena* | EU638114.1 |
|  | *hippurus* |  |  |  | *equiselis* |  | *hippurus* |  | *equiselis* |  |
| *Seriola dumerili* | *Seriola* | KY176627.1 |  |  | *Seriola* | EU036601.1 | *Seriola* | JX188874.1 | *Seriola* | JQ939167.1 |
|  | *dumerili* |  |  |  | *dumerili* |  | *dumerili* |  | *dumerili* |  |
| *Platax teira* | *Platax teira* | KU944946.1 |  |  | *Platax teira* | JQ937980.1 | *Platax teira* | EF539229.1 | *Platax teira* | JQ939140.1 |
| Gadidae sp. | *Merlangius*  *merlangus* | KX782666.1 | *Melanogrammus*  *aeglefinus* | AJ566336.2 | *Gadus morhua* | AF385832.1 | *--*.extitGadus morhua | EU002017.1 | *Gadus morhua* | DQ168066.1 |
| *Mola mola* | *Mola mola* | JF952793.1 |  |  | *Mola mola* | AF137215.1 | *Mola mola* | KF139644.1 | *Mola mola* | DQ168087.1 |
| *Diodon* sp. | *Diodon holo-* | MF041658.1 |  |  | *Diodon holo-* | KC442241.1 | *Diodon hys-* | EF539262.1 | *Diodon hys-* | KF027709.1 |
|  | *canthus* |  |  |  | *canthus* |  | *trix* |  | *trix* |  |
| *Ostracion* sp. | *Ostracion cu-* | KU945231.1 |  |  |  |  | *Ostracion cu-* | KF027638.1 | *Ostracion sp.* | DQ168095.1 |
|  | *bicus* |  |  |  |  |  | *bicus* |  |  |  |
