# Supplementary material for: Comparative Functional and Phylogenomic Analyses of Host Association in the Remoras (Echeneidae), a Family of Hitchhiking Fishes
Source: Integr Org Biol. 2019 May 10;1(1):obz007. doi: 10.1093/iob/obz007 (PMC7671162; doi:10.1093/iob/obz007)
Supplement: Supplementary_Table_obz007 [file supplementary_table_obz007.zip › Tables3.docx]

Table s3: Specimens analyzed in *µ*-CT analysis, their museum catalog numbers, and size in standard length (SL)

| Species | Cat. # | SL |
| --- | --- | --- |
| *Echeneis naucrates* | MCZ 30872 | 170 mm |
| *Echeneis naucrates* | USNM 202022 | 330 mm |
| *Echeneis naucrates* | MCZ 33306 | 180 mm |
| *Echeneis neucratoides* | USNM 348300 | 225 mm |
| *Echeneis neucratoides* | USNM 300452 | 330 mm |
| *Echeneis neucratoides* | MCZ 8678 | 200 mm |
| *Phtheirichthys lineatus* | USNM 326138 | 332 mm |
| *Phtheirichthys lineatus* | USNM 202100 | 315 mm |
| *Phtheirichthys lineatus* | MCZ 33448 | 145 mm |
| *Remora albescens* | MCZ 32104 | 94 mm |
| *Remora albescens* | MCZ 31364 | 60 mm |
| *Remora albescens* | MCZ 30798 | 42 mm |
| *Remora australis* | LACM 30310-18 | 182 mm |
| *Remora australis* | MCZ 8685 | 96 mm |
| *Remora australis* | CAS 26663 | 250 mm |
| *Remora brachyptera* | USNM 382506 | 245 mm |
| *Remora brachyptera* | USNM 202293 | 195 mm |
| *Remora brachyptera* | MCZ 8668 | 155 mm |
| *Remora osteochir* | MCZ 99592 | 195 mm |
| *Remora osteochir* | MCZ 101628 | 161 mm |
| *Remora osteochir*r | MCZ 43246 | 159 mm |
| *Remora remora* | MCZ40950 | 149 mm |
| *Remora remora* | MCZ83212 | 180 mm |
| *Remora remora* | MCZ83204 | 133 mm |
