# Supplementary material for: Comparative Functional and Phylogenomic Analyses of Host Association in the Remoras (Echeneidae), a Family of Hitchhiking Fishes
Source: Integr Org Biol. 2019 May 10;1(1):obz007. doi: 10.1093/iob/obz007 (PMC7671162; doi:10.1093/iob/obz007)
Supplement: Supplementary_Table_obz007 [file supplementary_table_obz007.zip › Tables4.docx]

Table s4: Surface roughness values of skin samples from select common hosts of the family Echeneidae. “Source” column indicates from where skin samples were obtained: Woods Hole Oceanographic Institute (WHOI), the Virginia Institute of Marine Sciences (VIMS), the Museum of Comparative Zoology (MCZ), and Boston Sword and Tuna (BST, a local commercial fish whole- saler). The samples acquired from the MCZ were preserved while the remainder of the samples acquired were recently dead or frozen specimens. All marine mammal samples were molded at the WHOI Marine Mammal Center

| Host species | *Sku* | *Sa* | source |
| --- | --- | --- | --- |
| *Carcharhinus falciformis* | 2.36 | 20.64 | MCZ |
| *Carcharhinus falciformis* | 2.92 | 10.66 | MCZ |
| *Carcharhinus falciformis* | 4.67 | 16.58 | MCZ |
| *Isurus oxyrinchus* | 3.02 | 10.47 | MCZ |
| *Isurus oxyrinchus* | 2.62 | 13.19 | MCZ |
| *Prionace glauca* | 3.05 | 21.00 | MCZ |
| *Prionace glauca* | 2.21 | 22.8 | MCZ |
| *Sphyrna zygaena* | 3.16 | 4.41 | MCZ |
| *Negaprion brevirostris* | 2.29 | 24.39 | MCZ |
| *Mobula birostris* | 3.87 | 25.06 | MCZ |
| *Mobula birostris* | 1.90 | 28.53 | MCZ |
| *Balaenoptera musculus* | 2.40 | 21.63 | WHOI |
| *Balaenoptera musculus* | 6.18 | 22.76 | WHOI |
| *Delphinus delphis* | 2.12 | 24.22 | WHOI |
| *Physeter macrocephalus* | 2.61 | 16.74 | WHOI |
| *Physeter macrocephalus* | 2.59 | 7.58 | WHOI |
| *Physeter macrocephalus* | 3.28 | 7.71 | WHOI |
| *Caretta caretta* | 1.92 | 3.36 | MCZ |
| *Caretta caretta* | 2.44 | 3.96 | MCZ |
| *Caretta caretta* | 2.03 | 7.56 | MCZ |
| *Megalops atlanticum* | 2.83 | 9.64 | MCZ |
| *Thunnus albacares* | 4.36 | 4.14 | MCZ |
| *Coryphaena* sp. | 3.20 | 3.90 | MCZ |

| *Istiophorus albicans* | 3.40 | 10.50 | MCZ |
| --- | --- | --- | --- |
| *Makaira nigricans* | 3.12 | 11.58 | VIMS |
| *Makaira nigricans* | 3.34 | 11.38 | VIMS |
| *Makaira nigricans* | 3.16 | 8.57 | VIMS |
| *Xiphias gladius* | 4.79 | 5.80 | BST |
| *Sphyraena barracuda* | 2.67 | 19.01 | MCZ |
| *Lactophrys quadricornis* | 4.11 | 54.50 | MCZ |
