# Supplementary material for: Comparative Functional and Phylogenomic Analyses of Host Association in the Remoras (Echeneidae), a Family of Hitchhiking Fishes
Source: Integr Org Biol. 2019 May 10;1(1):obz007. doi: 10.1093/iob/obz007 (PMC7671162; doi:10.1093/iob/obz007)
Supplement: Supplementary_Table_obz007 [file supplementary_table_obz007.zip › Tables5.docx]

Table s5: Remora host maximum length and speed values used to compute maximum host Reynolds number (*Re*). Maximum host lengths were retrieved from FishBase and SeaLifeBase. Maximum host swimming speeds were estimated using Domenici (2001)

| Host species | Max total length (m) | speed (m s*−*1) | *Re* |
| --- | --- | --- | --- |
| *Carcharias taurus* | 3.3 | 3.27 | 10338605 |
| *Carcharhinus falciformis* | 3.5 | 3.37 | 11285936 |
| *Carcharhinus leucas* | 3.6 | 3.41 | 11769741 |
| *Carcharhinus limbatus* | 2.8 | 2.99 | 7879196 |
| *Carcharhinus melanopterus* | 2.0 | 2.56 | 4902424 |
| *Carcharhinus obscurus* | 4.2 | 3.68 | 14808724 |
| *Ginglymostoma cirratum* | 4.3 | 3.72 | 15337133 |
| *Isurus oxyrinchus* | 4.5 | 3.79 | 16141081 |
| *Isurus paucus* | 4.3 | 3.71 | 15177971 |
| *Prionace glauca* | 4.0 | 3.59 | 13770369 |
| *Pseudocarcharias kamoharai* | 1.1 | 1.91 | 2011645 |
| *Negaprion brevirostris* | 3.4 | 3.32 | 10808857 |
| *Rhincodon typus* | 17.0 | 7.30 | 118917312 |
| *Rhizoprionodon acutus* | 1.8 | 2.40 | 4017935 |
| *Sphyrna zygaena* | 5.0 | 4.01 | 19201780 |
| *Triaenodon obesus* | 2.1 | 2.64 | 5384704 |
| *Istiophorus albicans* | 3.1 | 3.20 | 9646259 |
| *Delphinus capensis* | 2.5 | 2.85 | 6836075 |
| *Delphinus delphis* | 2.6 | 2.91 | 7247471 |
| *Physeter macrocephalus* | 24.0 | 8.65 | 198788355 |

| *Balaenoptera musculus* | 33.0 | 10.11 | 319493451 |
| --- | --- | --- | --- |
| *Caretta caretta* | 1.2 | 2.03 | 2433728 |
| *Alopias superciliosus* | 4.9 | 3.96 | 18518331 |
| *Makaira nigricans* | 5.0 | 4.01 | 19201780 |
| *Kajikia albida* | 3.0 | 3.12 | 8969885 |
| *Tetrapturus angustirostris* | 2.3 | 2.74 | 6037410 |
| *Kajikia audax* | 4.2 | 3.68 | 14808724 |
| *Tetrapturus belone* | 2.4 | 2.80 | 6432665 |
| *Xiphias gladius* | 4.5 | 3.83 | 16684499 |
| *Epinephelus itajara* | 2.5 | 2.85 | 6836075 |
| *Lutjanus apodus* | 0.7 | 1.50 | 965287 |
| *Scarus guacamaia* | 1.2 | 1.99 | 2290109 |
| *Scarus taeniopterus* | 0.3 | 1.09 | 365206 |
| *Sparisoma aurofrenatum* | 0.3 | 0.98 | 261904 |
| *Sparisoma chrysopterum* | 0.5 | 1.25 | 548764 |
| *Cheilinus undulatus* | 2.3 | 2.73 | 5998340 |
| *Cirrhilabrus rubripinnis* | 0.1 | 0.56 | 49074 |
| *Sparisoma viride* | 0.6 | 1.46 | 897603 |
| *Sphyraena barracuda* | 2.0 | 2.56 | 4902424 |
| *Acanthocybium solandri* | 2.5 | 2.85 | 6836075 |
| *Thunnus albacares* | 2.4 | 2.79 | 6392770 |
| *Thunnus obesus* | 2.5 | 2.85 | 6836075 |
| *Caranx ruber* | 0.6 | 1.41 | 795144 |
| *Caranx hippos* | 1.2 | 2.02 | 2404775 |
| *Platax teira* | 0.7 | 1.53 | 1025823 |
| *Masturus lanceolatus* | 3.4 | 3.30 | 10667060 |
| *Mola mola* | 3.3 | 3.29 | 10478958 |
| *Seriola dumerili* | 1.9 | 2.50 | 4541706 |
| *Mobula birostris* | 4.0 | 3.59 | 13770369 |
