# Supplementary material for: Comparative Functional and Phylogenomic Analyses of Host Association in the Remoras (Echeneidae), a Family of Hitchhiking Fishes
Source: Integr Org Biol. 2019 May 10;1(1):obz007. doi: 10.1093/iob/obz007 (PMC7671162; doi:10.1093/iob/obz007)
Supplement: Supplementary_Table_obz007 [file supplementary_table_obz007.zip › Tables6.docx]

Table s6: Eigenvector coefficients of phylogenetic PCA analysis and description of disc mor- phometric characters. For more detailed descriptions of morphometric variables see also See [Figure 2](#_bookmark59) and [Figure 2.](#_bookmark65) Lengths and width measurements were log_10_ transformed and divided by specimen standard length. Analysis was performed with mean values for each eight species of remora

PC1 PC2 PC3 variable description

disc AR -0.17 -0.55 -0.40 disc aspect ratio

disc area -0.60 -0.67 0.38 total disc area calculated as an ellipse (*π**length/2 *×* width/2) disc length -0.52 -0.78 0.33 disc length anteriormost to posteriormost margin of disc interc. AR -0.10 0.89 -0.13 aspect ratio of intercalary bone (width/length)

interc. cup width -0.75 -0.60 0.03 width of the intercalary bone depression

interc. process length -0.98 0.16 0.09 mean length of medial and lateral edges of intercalary bone process interc. process skew -0.26 -0.58 -0.61 skewness of intercalary bone processes (medial length/lateral length) int. ray head width -0.80 -0.56 -0.07 width of the head of the interneural ray

int. ray length -0.96 -0.08 0.20 length of the interneural ray

lam. AR -0.20 0.98 -0.02 aspect ratio of the lamella (width/length)

lam. angle -0.32 0.18 -0.45 aspect ratio of lamella (width/length) lam. process length -0.84 0.13 -0.43 length of the anteromedial process lam. count -0.04 -0.97 -0.02 number of lamella in disc system med. spinule length -0.96 0.00 -0.03 medial spinule length

ant. spinule length range -0.15 0.14 0.75 range of spinule length on anterior lamella

mid. spinule length range -0.20 0.04 0.92 range of spinule length on lamella at rostrocaudal midpoint post. spinule length range -0.27 0.21 0.77 range of spinule length on posteriormost lamella

ant. spinule density 0.90 -0.19 0.39 spinule density on anterior lamella (n mm*−*2)

mid. spinule density 0.83 -0.24 0.26 spinule density lamella at rostrocaudal midpoint (n mm*−*2) post. spinule density 0.90 -0.26 0.27 spinule density on posteriormost lamella (n mm*−*2)

ant. spinule mean length -0.80 0.45 -0.09 mean spinule length on anterior lamella

mid. spinule mean l ength -0.93 0.26 0.23 mean spinule length on lamella at rostrocaudal midpoint post. spinule mean length -0.90 0.19 0.
