# Supplementary material for: Comparative Functional and Phylogenomic Analyses of Host Association in the Remoras (Echeneidae), a Family of Hitchhiking Fishes
Source: Integr Org Biol. 2019 May 10;1(1):obz007. doi: 10.1093/iob/obz007 (PMC7671162; doi:10.1093/iob/obz007)
Supplement: Supplementary_Table_obz007 [file supplementary_table_obz007.zip › Tables7.docx]

Table s7: Summary of mean phylogenetic distance with standardized effect size (MPD obs. z) analysis of echeneid hosts

|  | Host  number | MPD obs. | MPD rand.  mean | MPD  rand. SD | MPD  rank | obs. | MPD  z | obs. | MPD obs.  p |
| --- | --- | --- | --- | --- | --- | --- | --- | --- | --- |
| *E. naucrates* | 40 | 505.37 | 573.74 | 27.46 | 16.00 |  | -2.49 |  | 0.02 |
| *E. neucratoides* | 3 | 605.29 | 566.66 | 210.03 | 383.00 |  | 0.18 |  | 0.38 |
| *P. lineatus* | 9 | 609.22 | 579.06 | 87.55 | 550.00 |  | 0.34 |  | 0.55 |
| *R. albescens* | 2 | 530.00 | 564.91 | 323.28 | 442.00 |  | -0.11 |  | 0.44 |
| *R. australis* | 6 | 41.17 | 576.34 | 117.67 | 1.00 |  | -4.55 |  | *<*0.01 |
| *R. brachyptera* | 15 | 547.07 | 573.26 | 62.51 | 285.00 |  | -0.42 |  | 0.28 |
| *R. osteochir* | 12 | 281.46 | 575.65 | 69.06 | 2.00 |  | -4.26 |  | *<*0.01 |
| *R. remora* | 24 | 574.14 | 574.87 | 43.42 | 438.00 |  | -0.02 |  | 0.44 |
